# Voice attractiveness and decision making in third‐party punishment

**DOI:** 10.1002/pchj.690

**Published:** 2023-10-31

**Authors:** Junchen Shang, Zhihui Liu, Chang Hong Liu

**Affiliations:** ^1^ Department of Medical Humanities, School of Humanities Southeast University Nanjing China; ^2^ College of Psychology, Liaoning Normal University Dalian China; ^3^ Department of Psychology Bournemouth University Poole UK

**Keywords:** beauty premium, dictator game, perceived reasonableness, third‐party punishment, voice attractiveness

## Abstract

This study examined the impact of an attractive voice compared to an unattractive voice in an economic game. Results showed that proposers with an attractive voice were perceived as more reasonable in their monetary allocations and were less likely to receive punishment for unfair allocation.

## INTRODUCTION

In economic games, participants are more willing to accept unfair offers from proposers with an attractive voice (Shang et al., 2021; Shang & Liu, 2022a, 2022b). This effect seems to be stronger when the voice lasts for a longer period of time (2040 ms) than a shorter one (400 ms) (Shang & Liu, 2022a, 2022b). However, since human cooperation involves both fair exchange and punishment of violators of social norms (Fehr & Gächter, 2002), it is essential to know whether effects of voice attractiveness also extend to punishing behaviors. Here we investigated this question in a modified dictator game (Li & Zhou, 2014), where participants acted as an interest‐free third party who were not involved in financial allocation.

## METHODS

A total of 111 students (*M*_age_ = 21.47 years, *SD* = 2.17, 57 females) from Liaoning Normal University were randomly assigned to two voice conditions. The research was approved by the Institutional Review Board of Liaoning Normal University. Written informed consent was obtained from each participant.

This was a 2 (duration of voice: 2040, 400 ms) × 2 (voice attractiveness: attractive, unattractive) × 3 (fairness: fair, mildly unfair, unfair) mixed factorial design, where duration of voice was a between‐participant factor, whereas voice attractiveness and fairness were within‐participant factors. Fairness was defined by how much out of ¥100 the proposer allocated to the recipient. Allocations of ¥50 or ¥40 were considered fair, ¥30 was considered mildly unfair, and ¥10 or ¥20 were considered unfair. The stimuli were 36 attractive and 36 unattractive 400‐ms voices and 24 attractive and 24 unattractive 2040‐ms voices (Shang & Liu, 2022a, 2022b). Each 400‐ms voice pronounces one of five nonsense syllables (/a/, /ai/, /ao/, /ei/, /ou/), and each 2040‐ms voice pronounces three nonsense syllables (/i/, /a/, /ou/).

Participants were told that they would hear a proposer's voice and only the proposer could make an allocation to split ¥100 among the proposer himself/herself and the recipient, who had to accept it. In each trial, a photo of a ¥100 banknote was displayed for 500 ms in the center of the screen followed by a 1000 ms fixation cross. A proposer's voice was then presented for 400 ms in one group, but 2040 ms in another. At the onset of the voice, the monetary allocation was displayed on the screen for 2500 ms. The reasonableness scale was presented following the allocation screen. The participants rated the reasonableness of the monetary division by clicking the mouse button (on a 9‐point scale from −4 = “very unreasonable” to 4 = “reasonable”). They then indicated the extent to which they wanted to punish the proposer by clicking the mouse button on a 9‐point scale from 0 (not at all) to 8 (very much). In the 2040‐ms condition, 16 voices were paired with each fairness level. Each pair of voice and allocation was repeated three times. In the 400‐ms condition, 24 voices were paired with each fairness level. Each pair of voice and allocation was repeated twice. Half the voices in each allocation were attractive, the other half were unattractive. This amounted to a total of 144 experimental trials for each voice duration condition. The presentation of the experimental trials was random. After the experiment, the participants rated the attractiveness of the voices on a 7‐point scale (from 1 = “unattractive” to 7 = “attractive”).

## RESULTS

The agreement of the ratings across repetition for all voice duration conditions was high (all Cronbach's *α* ≥ .95).

**FIGURE 1:**
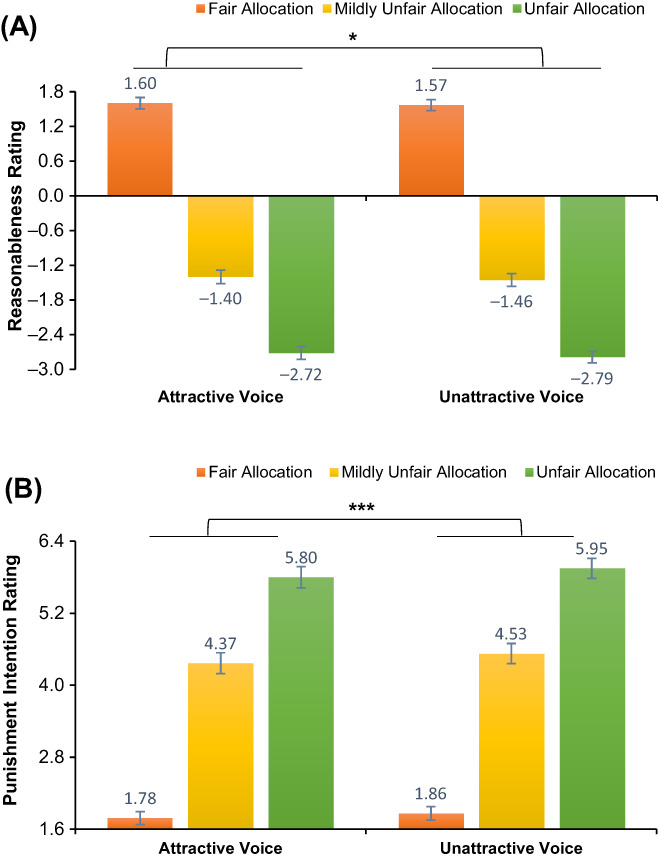
(A) Mean reasonableness rating as a function of the fairness of allocation and proposers' voice attractiveness. (B) Mean punishment intention rating as a function of the fairness of allocation and proposers' voice attractiveness. The error bars represent standard errors. *p < .05, ***p < .001.

Three‐way ANOVAs on the two rating scores found no main effect of voice duration or interaction of this variable with other variables, hence we collapsed this variable in subsequent analyses. Greenhouse–Geisser correction was applied for sphericity departures. Results for reasonableness rating (Figure 1A) showed that allocations from proposers associated with attractive voices were rated as more reasonable than those with unattractive voices, *F*(1, 109) = 5.52, *p* = .021, *η*_p_^2^ = .05. The main effect of fairness was also significant, *F*(1.34, 146.33) = 874.90, *p* < .001, *η*_p_^2^ = .89, where fair allocations were rated as more reasonable than mildly unfair and unfair allocations, and mildly unfair allocations were rated as more reasonable than unfair allocations, *p*s < .001. There was no interaction between the two variables.

Results for punishment intention rating (Figure 1B) showed that participants were more willing to punish proposers associated with unattractive voices than those with attractive voices, *F*(1, 109) = 14.95, *p* < .001, *η*_p_^2^ = .12. The main effect of fairness was also significant, *F*(1.57, 170.91) = 548.50, *p* < .001, *η*_p_^2^ = .83, where participants were more willing to punish proposers who made unfair allocations than those who made mildly unfair or fair allocations, and more willing to punish proposers who made mildly unfair allocations than those who made fair allocations, *p*s < .001. No interaction was found between the two variables.

Finally, the results of the attractiveness rating confirmed that attractive voices (*M* = 5.10, *SD* = 0.44) were rated as more attractive than unattractive voices (*M* = 2.84, *SD* = 0.57), *F*(1, 116) = 559.84, *p* < .001, *η*_p_^2^ = .83.

## DISCUSSION

Our findings demonstrate that the attractiveness of a proposer's voice can affect the decision to punish unfair allocation in an economic game. Allocations by proposers with an attractive voice were less likely to be punished when they made unfair allocations. They were also judged as more reasonable than those with an unattractive voice. These findings are consistent with previous studies that also revealed a beauty premium effect of voice attractiveness (Shang et al., 2021; Shang & Liu, 2022a, 2022b). However, while participants' decisions in the past studies had financial consequences riding on them, the present study shows that similar effects can be observed even when participants are third parties who are not involved in the monetary payoff. We should note, however, that the effect of attractiveness had a much smaller effect size than the effect of fairness.

Prior research has shown that the effect of voice attractiveness was stronger for a longer voice duration than a shorter one (Shang & Liu, 2022a, 2022b). However, the effect of the two durations was the same in the present study. One potential reason for this discrepancy is whether monetary payoffs to participants were involved in the game. While the prior research used a trust game in which participants' gain and loss of money induced different event‐related potentials, the present study used a dictator game in which participants acted as interest‐free third parties and observed the proposer's monetary allocations. The between‐subject design could also have made it more difficult to detect a small difference. Finally, we should note that because only nonsense syllables were used in this experiment, future research should use real words to determine whether word valence interacts with voice attractiveness.

## CONFLICT OF INTEREST STATEMENT

The authors declare there are no conflicts of interest.

## ETHICS STATEMENT

The research was approved by the Institutional Review Board of Liaoning Normal University. Written informed consent was obtained from each participant.
